# Abbreviated Half-Lives and Impaired Fuel Utilization in Carnitine Palmitoyltransferase II Variant Fibroblasts

**DOI:** 10.1371/journal.pone.0119936

**Published:** 2015-03-17

**Authors:** Min Yao, Min Cai, Dengfu Yao, Xi Xu, Rongrong Yang, Yuting Li, Yuanyuan Zhang, Hiroshi Kido, Dengbing Yao

**Affiliations:** 1 School of Life Sciences, Key Laboratory of Neuroregeneration, Co-innovation Center of Neuroregeneration, Nantong University, Nantong, Jiangsu, P. R. China; 2 School of Medicine, Affiliated Hospital of Nantong University, Nantong, Jiangsu, P. R. China; 3 Division of Enzyme Chemistry, Institute for Enzyme Research, the University of Tokushima, Tokushima, Japan

## Abstract

Carnitine palmitoyltransferase II (CPT II) deficiency is one of the most common causes of fatty acid oxidation metabolism disorders. However, the molecular mechanism between *CPT2* gene polymorphisms and metabolic stress has not been fully clarified. We previously reported that a number of patients show a thermal instable phenotype of compound hetero/homozygous variants of CPT II. To understand the mechanism of the metabolic disorder resulting from CPT II deficiency, the present study investigated CPT II variants in patient fibroblasts, [c.1102 G>A (p.V368I)] (heterozygous), [c.1102 G>A (p.V368I)] (homozygous), and [c.1055 T>G (p.F352C)] (heterozygous) + [c.1102 G>A (p.V368I)] (homozygous) compared with fibroblasts from healthy controls. CPT II variants exerted an effect of dominant negative on the homotetrameric proteins that showed thermal instability, reduced residual enzyme activities and a short half-life. Moreover, CPT II variant fibroblasts showed a significant decrease in fatty acid β-oxidation and adenosine triphosphate generation, combined with a reduced mitochondrial membrane potential, resulting in cellular apoptosis. Collectively, our data indicate that the CPT II deficiency induces an energy crisis of the fatty acid metabolic pathway. These findings may contribute to the elucidation of the genetic factors involved in metabolic disorder encephalopathy caused by the CPT II deficiency.

## Introduction

The carnitine palmitoyl-transferase (CPT) system is component of adenosine triphosphate (ATP) production through fatty acid β-oxidation [1–7]. It consists of two enzymes CPT I and CPT II which located in the mitochondrial membranes. The CPT system transfers long chain fatty acids from cytosol to mitochondrial. The rate-limiting step in the transfer of long chain fatty acid is that CPT I transesterificates acyl-CoA to acylcarnitine [8, 9], but CPT II changes acylcarnitine to acyl-CoA. The CPT II protein is a homotetramer encoded by *CPT2* gene which located on chromosome 1p32, contains five exons ranging from 81 to 1,305 bp in size. Fatty acid metabolized by β-oxidation pathway represents energy source performed particular in muscle and also in nerve system. The autonomic recessive CPT II deficiency is the inherited disease of the mitochondrial long chain fatty acid oxidation, includes a severe infantile hepatocardiomuscular form, a lethal neonatal form, and a myopathic form [10–14].

To date, more than 70 different mutations of *CPT2* have recently been reported, and studies have demonstrated the relationships between the CPT II deficiency genotypes and their clinical phenotypes characterized by febrile convulsions and multiple-organ failure during high fever [15–21]. However, the molecular mechanism underlying the relationship between *CPT2* polymorphisms and metabolic stress has not been completely clarified. Previously, we have reported that a number of influenza-associated encephalopathy patients exhibit a thermolabile phenotype of compound hetero/homozygous variants of CPT II [20–22], and analyzed the enzymatic properties of CPT II variants expressed in transfected COS-7 cells. The direct biochemical consequences of the mutations are still controversial. To understand the mechanisms of the metabolic disorder resulting from CPT II deficiency, we studied CPT II variants in patient fibroblasts, [c.1102 G>A (p.V368I)] (heterozygous), [c.1102 G>A (p.V368I)] (homozygous), and [c.1055 T>G (p.F352C)] (heterozygous) + [c.1102 G>A (p.V368I)] (homozygous) compared with control fibroblasts. Our study hypothesis was that the CPT II deficiency caused a lack of enzymatically active CPT II protein and induced an energy crisis in the fatty acid metabolic pathway. Our findings may contribute to the elucidation of the genetic factors involved in metabolic disorder encephalopathy.

## Materials and Methods

### Materials

[1-^14^C] oleic acid, L-[methyl-^3^H] carnitine, L-[^35^S] methionine, and enhanced chemiluminescence detection reagents were bought from Amersham Pharmacia Biotech (Piscataway, NJ, USA), the BCA reagent was from Pierce (Rockford, IL, USA). The ABI DyeDeoxy Terminator Cycle Sequencing Kit was obtained from PE-Applied Biosystems (Foster City, CA, USA).

### CPT II deficiency patients and *CPT2* polymorphism analysis

This study was approved by the Ethics Review Committee for human genome analysis of the Key Laboratory of Neuroregeneration, Nantong University. Informed written consents were obtained from the adult patients and from the next of kin, caretakers, or guardians on behalf of the minor patients in this study. To diagnose CPT II deficiency, patient plasma was examined to determine the accumulation of long-chain acylcarnitines, which is indicative of disease. A total of 12 patients were diagnosed with CPT II deficiency on the basis of clinical manifestations, including the abrupt onset of seizures and coma developing within 12–48 h after the onset of high fever (≥40°C). In all patients, the age range was from 1 to 56 years, eight patients were males and four were females. To confirm the diagnosis, *CPT2* mutation analysis was performed by PCR-amplifying the five coding exons and flanking intronic regions of *CPT2* from genomic DNA extracted from EDTA—whole blood. The primers were intronic (S1 Table), and have been described previously [20–23]. PCR products were sequenced directly by using ABI-PRISM 3100 Genetic Analyzer. The PCR products were sequenced in both strands and the analysis were performed in triplicate. The sequencing results showed the following mutations: NM_000098.2 (CPT2_v001): [c.1102 G>A (p.V368I)] (heterozygous), and [c.1102 G>A (p.V368I)] (homozygous), [c.1055 T>G (p.F352C)] (heterozygous) + [c.1102 G>A (p.V368I)] (homozygous).

### Assay of CPT II activity and kinetic properties

We collected the fibroblasts of CPT II variations with clinical symptoms and biochemical markers characteristic of this inherited metabolic disorder. The control fibroblasts were bought from company. These fibroblasts were cultured at 37°C in 5% CO_2_ in Dulbecco’s modified Eagle’s medium (DMEM) containing 10% fetal serum (Indianapolis, IN, USA). CPT II activities in the fibroblast lysates were measured at 30°C for 2 h, by detection of palmitoyl-L-[methyl-^3^H] carnitine formed from L-[methyl-^3^H] carnitine and palmitoyl-CoA [6]. The palmitoyl-L-[methyl-^3^H] carnitine formed was extracted and radioactivity levels were counted. The *V*_max_ and *K*_m_ values were analyzed by measuring the enzyme activity at 30°C by varying the L-[methyl-^3^H] carnitine concentrations. To analyze the heat stability of control and CPT II deficiency fibroblasts, cell lysates were pre-incubated at conditions simulating a high fever (41°C) or stable conditions (30°C) for 0–2 h, and the enzyme activities were assayed after the addition of 200 μM L-[methyl-^3^H] carnitine then continue incubated at 30°C for 2 h [20–23]. The protein concentrations in the fibroblast lysates were assayed using the bicinchoninic acid protein assay reagent kit.

### Expression of control and patient *CPT2* mRNA

To analyze *CPT2* mRNA expression levels of control and patient fibroblasts, total RNA was extracted using the RNeasy Mini Kit. The fibroblasts cDNA were synthesized using a random primer and reverse transcriptase. The expression of *CPT2* mRNA levels was analyzed by real-time quantitative PCR with *CPT2*-specific primers.

CPT II protein expression of control and patient fibroblasts was analyzed by sodium dodecyl sulfate polyacrylamide gel electrophoresis (SDS-PAGE) under reducing conditions, followed by Western Blotting with anti-CPT II antibodies. The amount of CPT II total RNA and proteins were expressed relative to those of β-actin control.

### Mitochondrial membrane potential analysis

Fibroblasts were cultured for 5 h at 37°C or 41°C and then continue incubated for an another 15 min with 5,5′, 6,6′-Tetrachloro-1,1′,3,3′-tetraethylbenzimidazolyl-carbocyanineiodide fluorescent dye (JC-1) from the Probes in the dark. Then they were washed with assay buffer and immediately imaged with the red fluorescence channel (λ_excitation_: 560 ± 40 nm band pass filter, λ_detection_: 630 ± 60 nm band pass filter) using a fluorescence microscope and with the green fluorescence channel (λ_excitation_: 470 ± 40 nm band pass filter, λ_detection_: 535 ± 50 nm band pass filter) [20, 21].

### Apoptosis analysis of CPT II variants

The percentage of apoptotic control and patient fibroblasts was measured by flow cytometry. Annexin V—FITC was added to cell suspensions following propidium iodide labelling, and the number of apoptotic fibroblasts was counted by FACScan flow cytometry according to the manufacturer’s protocol and based on an annexin V flow cytometry histogram. Fibroblasts incubated with dimethyl sulfoxide (DMSO) were used as a control. Control and patient fibroblasts were also analyzed by a lactate dehydrogenase (LDH) cytotoxicity assay. The LDH reagent and catalyst was added to the supernatant from fibroblast medium and incubated for 30 min at room temperature. The absorbance was recorded at 490 nm with background subtraction at 630 nm using the Synergy HT multi-detection Micro Plate Reader, and results were shown as a percentage of LDH release. To analyze the apoptotic factor release, fibroblasts were lysed and immunoblotted separately with anti-caspase-3,-caspase-8,-cytochrome c, and-Bid antibodies. CPT II protein expression levels were expressed relative to those of β-actin. All experiments were carried out in triplicate.

### CPT II half-lives of control and patient fibroblasts

Cultured fibroblasts were washed with PBS and incubated for 48 h in serum- and methionine-free medium 30 min, then followed by pulse chase labeling with [^35^S]-methionine (specific activity 1,000 Ci/mM) for 2 h. After pulse labeling, the fibroblasts were again washed with PBS and chased for 0, 6, 12, and 18 h. At each chase interval, the fibroblasts were lysed and immunoprecipitated with anti-CPT II antibody coupled to Protein G-Sepharose 4B. Then the immunoprecipitates were separated by SDS-PAGE and the dried gels were analyzed autoradiographically [20, 21].

### Mitochondrial fatty acid β-oxidation and ATP generation

The β-oxidation of mitochondrial fatty acids was analyzed as previously described by Saudubray et al. [20]. Control and patient fibroblasts were cultured at 37°C or 41°C in plastic center containing a filter paper, then continue cultured for an additional 2 h. At the end of the incubation, the reaction was stopped by the addition of perchloric acid, and the radioactive CO_2_ released from the fibroblasts was collected for 1 h at room temperature by injecting NaOH onto the filter paper under slow shaking. The filter papers were retrieved and the amount of ^14^CO_2_ radioactivity was counted using liquid scintillation counting in ACS II in a Beckman LS 6500 multi-purpose scintillation counter [20].

Fibroblasts were washed twice with PBS and cellular ATP was extracted with 1% trichloroacetic acid, then the ATP production concentration was measured. The extracts were centrifuged and immediately diluted with 1 M Tris-acetate buffer. The total ATP generation in the extracts was measured by an ATP assay system bioluminescence detection kit according to the manufacturer’s instructions.

### Statistical analysis

The data were statistical analyzed using SPSS 15.0 for windows. The Student’s t-test was used for comparison between the groups. P<0.05 was considered statistically significant. All data were expressed as mean ± SD.

## Results

### *CPT2* polymorphisms in CPT II deficiency patients

Genotype analyses of *CPT2* in patients suffering from CPT II deficiency identified three polymorphisms associated with amino acid substitutions: [c.1102 G>A (p.V368I)] (heterozygous), [c.1102 G>A (p.V368I)] (homozygous), and [c.1055 T>G (p.F352C)] (heterozygous) + [c.1102 G>A (p.V368I)] (homozygous). These variations matched the mutations previously reported in late-onset muscular, infantile/juvenile hepatic, and severe neonatal phenotypes of CPT II deficiency [24–30]. Mutations [c.1055 T>G (p.F352C)] (heterozygous) + [c.1102 G>A (p.V368I)] (homozygous) are located near the acylcarnitine binding residues [26], and transient serum acylcarnitine ratios [(C_16:0_ + C_18:1_)/C_2_] of >0.09 were previously observed during high fever. These variations were found not only in disease-causing mutations defined as pathogenic but also found in polymorphisms of *CPT2* gene associated with inherited CPT II deficiency. The three polymorphisms found in *CPT2* gene and in the studied patients [c.1102 G>A (p.V368I)] (heterozygous), [c.1102 G>A (p.V368I)] (homozygous), and [c.1055 T>G (p.F352C)] (heterozygous) + [c.1102 G>A (p.V368I)] (homozygous) matched the mutations previously reported in late-onset muscular, infantile/juvenile hepatic, and severe neonatal phenotypes of CPT II deficiency.

### Enzymatic properties and thermal instability of CPT II variants

We next examined the CPT II activity and kinetic properties of fibroblast lysates. The CPT II activity of control fibroblasts was 0.28 ± 0.05 nmol/min/mg protein (n = 5), that of p.V368I (homozygous) was 0.27 ± 0.05 nmol/min/mg protein (n = 5), p.F352C (homozygous) was 0.18 ± 0.05 nmol/min/mg protein (n = 5), and p.F352C + p.V368I was 0.15 ± 0.05 nmol/min/mg protein (n = 5) (S2 Table). These results are similar to those reported in a previous study [20–23].

**Fig 1:**
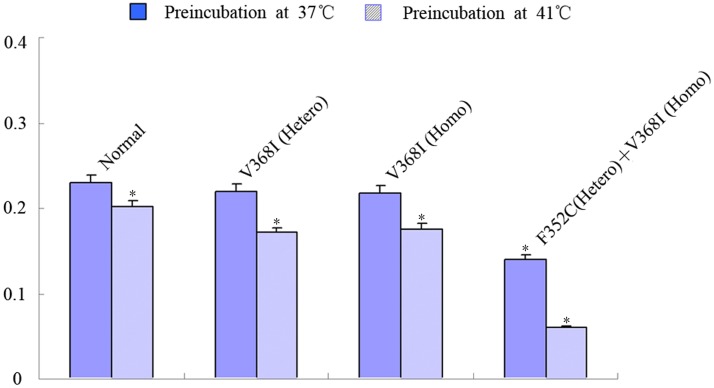
CPT II activities and thermal instability in CPT II-deficient fibroblasts. Fibroblast lysates were preincubated at 37°C or 41°C. Enzymatic reactions commenced by the addition of substrates at 37°C. Data are the means of five separate experiments. The average of three independent experiments is shown ± SEM (*P<0.05).

We previously reported that CPT II deficiency is a thermolabile phenotype of compound hetero-/homozygous variants [20–23], so we next evaluated the temperature sensitivity of CPT II variants. Fibroblast lysates were pre-incubated at 41°C or 30°C for 0–2 h, and then CPT II activities were measured at 30°C following the addition of L-carnitine and palmitoyl-CoA substrates (Fig. 1). Of the three variants, p.F352C + p.V368I was the most unstable, with significantly reduced enzyme activities during incubation at 41°C; under the same conditions, the activities of control fibroblasts and those from variants p.V368I (heterozygous) and p.V368I (homozygous) remained low and were also markedly reduced.

### CPT II expression and dominant—negative effect

**Fig 2:**
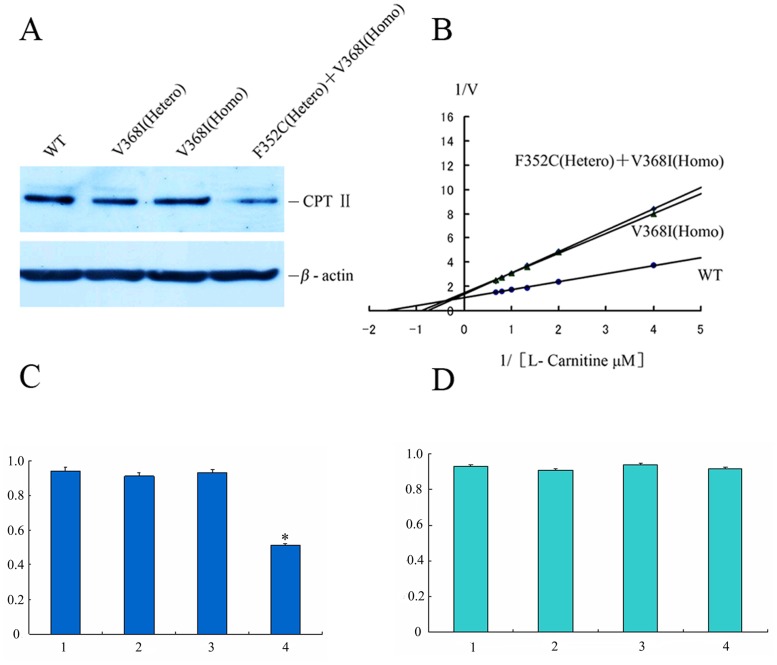
CPT II expression and the dominant—negative effect of *CPT II* variants on substrate-dependent kinetics. (A) CPT II expression was analyzed by Western Blotting with an anti-CPT II antibody. (B) Dominant—negative effect of *CPT II* variants was analyzed by substrate-dependent kinetics. (C) Relative protein expression of control and variant CPT II. (D) Real-time PCR analysis of control and variant *CPT II* expression. Lane 1, control fibroblasts; lane 2, V368I (heterozygous); lane 3, V368I (homozygous); lane 4, F352C (heterozygous) + V368I (homozygous). CPT II protein was expressed relative to β-actin. *V*_max_ and *K*_m_ values were obtained from kinetic analysis (1/V versus 1/[S] plots) by varying the concentrations of L-[methyl-^3^H] carnitine between 0–300 μM at a fixed 50 μM palmitoyl-CoA concentration. (●) control, (▲) V368I (Homo), (◆) F352C (Hetero) + V368I (Homo). Data are means of three separate experiments. The average of three independent experiments is shown ± SEM (*P<0.05)

Previous studies of enzyme activity in fibroblasts of CPT II-deficient patients revealed similar CPT II activities for homozygous and compound heterozygous *CPT2* mutations [19], suggesting that mutant *CPT2* exert a dominant negative effect on the CPT II protein [31]. We therefore further examined the *V*_max_ and *K*_m_ values for L-carnitine of CPT II variant fibroblasts. *V*_max_ and *K*_m_ values of p.V368I (homozygous) were similar to those of p.F352C (heterozygous) + p.V368I (homozygous) (Fig. 2B). These data confirm an effect of homozygous and heterozygous *CPT2* mutations on the enzymatic properties of the homotetrameric protein compared with those of control CPT II.

To evaluate the stability of these variant CPT IIs, we analyzed CPT II mRNA and protein expression in control and patient fibroblasts. CPT II protein expression levels of p.F352C + p.V368I were significantly lower than p.V368I and p.V368I compared the CPT II protein expression levels of p.F352C +p.V368I with p.V368I and p.V368I according to Western Blotting (Fig. 2A, 2C), although mRNA levels, as measured by real-time PCR, were comparable (Fig. 2D). This indicates that the functional disorder of CPT II deficiency does not compromise the synthesis of polypeptides, but instead appears to impair the intracellular stability of folded CPT II proteins.

### Short half-lives of CPT II variants

**Fig 3:**
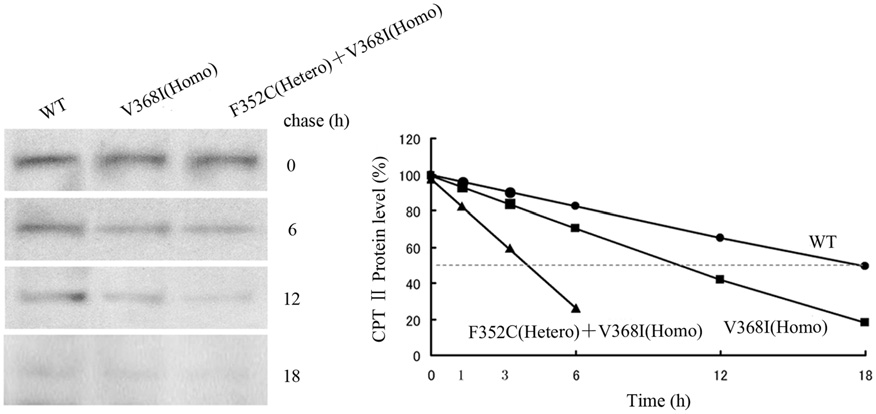
Pulse-chase (left) and half-lives (right) of control and variant CPT II in fibroblasts. Cultured fibroblasts were pulse-labeled with L-[^35^S] methionine for 2 h and chased for 0, 6, 12, and 18 h. CPT II from fibroblast lysates was immunoprecipitated with anti-CPT II antibodies, then subjected to SDS-PAGE followed by autoradiography. (●) control, (■) V368I (homozygous), (▲) F352C (heterozygous) + V368I (homozygous). The average of three independent experiments is shown ± SEM.

To examine the intracellular stability of CPT II variants, we metabolically labeled control and patient fibroblasts with L-[^35^S] methionine and measured their half-lives using a pulse-chase protocol (Fig. 3, left). No differences were detected in the concentrations of newly synthesized and labeled CPT II proteins between the immunoprecipitates of control and patient fibroblasts, suggesting that the synthesis of control and patient CPT IIs was similarly efficient. Time course experiments showed that control CPT IIs had the longest half-life (T^1/2^ ~ 18 h), similar to our previous data, while the half-life of p.V368I (homozygous) was shorter at T^1/2^ ~ 10 h, and that of p.F352C (heterozygous) + p.V368I (homozygous) was significantly shorter, at T^1/2^ ~ 4 h (Fig. 3, right).

### Reduction of mitochondrial membrane potential

**Fig 4:**
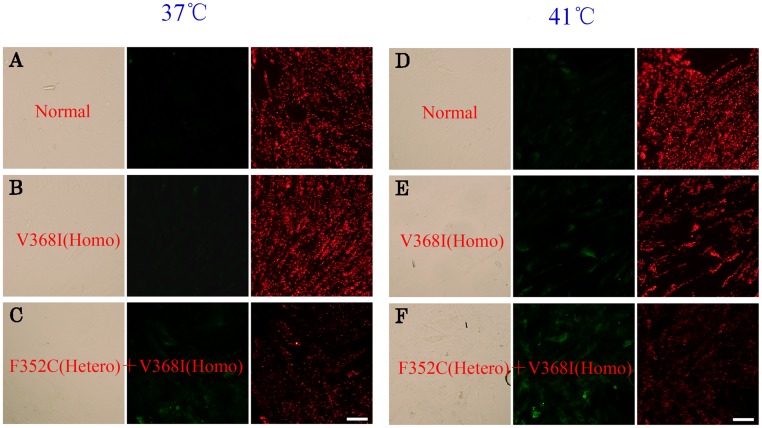
Reduction of mitochondrial membrane potential of control and variant CPT IIs in cultured fibroblasts. Control (A, D), V368I (homozygous) (B, E), and F352C (heterozygous) + V368I (homozygous) (C, F) fibroblasts were cultured at 37°C and 41°C. Mitochondrial depolarization was monitored by 15 min treatment with 10 μM of JC-1 in the dark and visualized under a fluorescence microscope. Scale bars, 100 μm.

We assessed the mitochondrial membrane potential (ΔΨ_m_) using the cationic lipophilic probe JC-1. Normal mitochondria with a high ΔΨ_m_ appear red following aggregation of JC-1, which emits red fluorescence at ~590 nm. Following mitochondrial depolarization with a low ΔΨ_m_, the JC-1 dye remains in its monomeric form, thereby emitting relatively more green (~525 nm) fluorescence [32]. We observed an increase of green fluorescence and a reduction of red fluorescence in the mitochondria of fibroblasts from patients with p.V368I (homozygous) and p.F352C (heterozygous) + p.V368I (homozygous) variants compared with mitochondria from control fibroblasts at both 37°C (Fig. 4A, B, C) and 41°C (Fig. 4D, E, F). Mitochondrial depolarization is a marker of mitochondrial dysfunction and precedes ATP depletion, so the data indicate a relatively low ΔΨ_m_ of patient fibroblasts at 37°C and an enhanced decrease in ΔΨ_m_ under heat-stress conditions at 41°C.

### Cell apoptosis of CPT II variants

**Fig 5:**
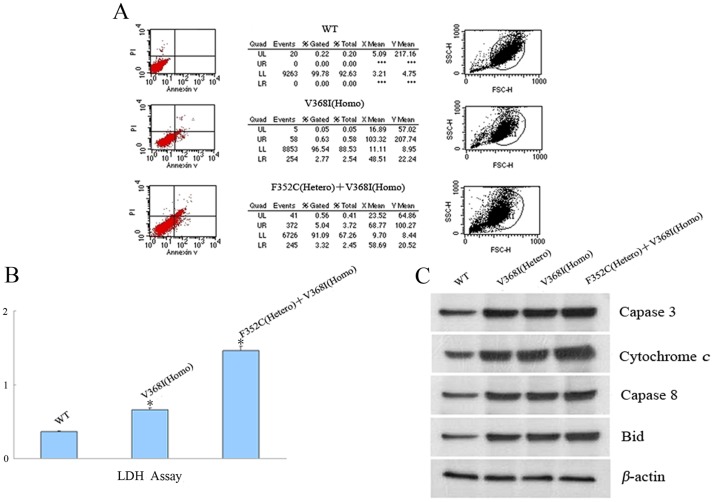
Cell apoptosis analysis of *CPT II* variants in fibroblasts. (A) Apoptotic fibroblasts were measured by flow cytometry. Fibroblasts incubated with DMSO were used as a control. (B) LDH assay of control and *CPT II* variants in fibroblasts. (C) Apoptotic factor release was analyzed by Western Blotting with antibodies against caspase-3, caspase-8, cytochrome c, and Bid for control and variant CPT IIs in control and CPT II-deficient fibroblasts. CPT II proteins are expressed relative to β-actin. Data are means of three separate experiments. The average of three independent experiments is shown ± SEM (*P<0.05).

To assay cell apoptosis induced by CPT II deficiency, the percentage of apoptotic fibroblasts was measured in control and patient fibroblasts. Flow cytometry analysis revealed a significant increase in apoptosis of cultured patient fibroblasts from p.V368I (homozygous) and p.F352C (heterozygous) + p.V368I (homozygous) variants compared with control fibroblasts (Fig. 5A).

LDH is a stable cytoplasmic enzyme that catalyzes the interconversion of lactate and pyruvate. It is ubiquitously expressed in all cells and is rapidly released into the supernatant upon the plasma membrane damage. Therefore, measurement of its activity can be used to quantify plasma membrane damage. We observed a significant increase, seen as a percentage of LDH release, in damage to cultured patient fibroblasts p.V368I (homozygous) and p.F352C (heterozygous) + p.V368I (homozygous) (Fig. 5B).

To further evaluate the apoptotic effect in CPT II variants, the expression of apoptosis factors was analyzed by immunoblotting. Caspases are responsible for the deliberate of cells into apoptotic bodies during cell apoptosis. Caspases 3 and caspases 8 are situated at important junctions in apoptosis pathways, and activate disassembly in response to agents or insults that trigger the cytochrome c release from mitochondria. Caspase 3 amplifies caspase 8 initiation signals into a commitment to disassembly, while caspase 8 activates caspase 3 by proteolytic cleavage and cleaves vital cellular proteins. In the present study, Western Blotting showed that apoptotic factor was significantly released (Fig. 5C). Moreover, caspase-3, caspase-8, cytochrome c, and Bid, which induces apoptosis and allows the release of cytochrome c, were all expressed at higher levels in fibroblasts from p.V368I (homozygous) and p.F352C (heterozygous) + p.V368I (homozygous) variants compared with control fibroblasts.

### Correlation between CPT II activity and ATP generation

**Fig 6:**
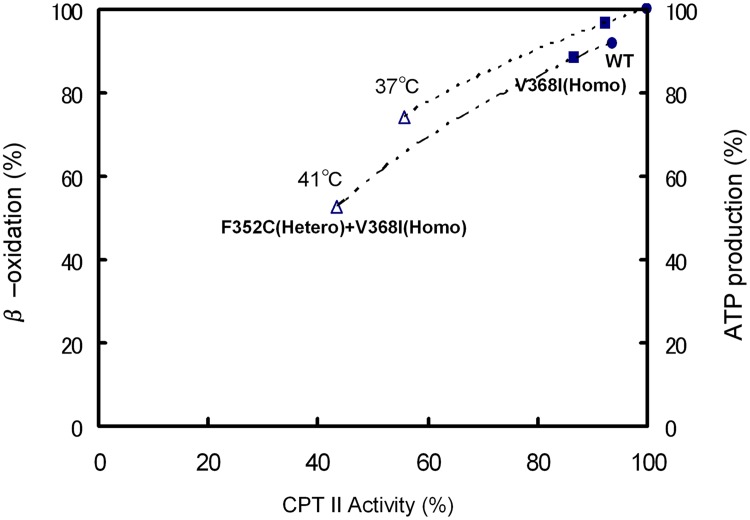
Correlation between CPT II activity and β-oxidation/ATP production at 41°C and 37°C in CPT II-deficient fibroblasts. (●) control, (■) V368I (homozygous), (△) F352C (heterozygous) + V368I (homozygous). CPT II activity, β-oxidation, and ATP production are expressed as % of control values of control fibroblasts at 37°C. Data areand means of five separate experiments: 0.53 nmol/min/mg protein of CPT II activity; 14.5 nmol of released CO_2_/h/10^6^ cells; 8.12 nmol of ATP/10^6^ cells. The average of three independent experiments is shown ± SEM.

To further clarify the metabolic disorder resulting from CPT II deficiency, the effects of thermal instability, CPT II enzymatic activities (S2 Table), fatty acid oxidation flux, and cellular ATP production were analyzed at 37°C and 41°C in patient fibroblasts p.V368I (homozygous), p.F352C (heterozygous) + p.V368I (homozygous) and control fibroblasts (Fig. 6). CPT II activities, ATP production, fatty acid oxidation and of fibroblasts from patients with the p.V368I (homozygous) variant were decreased to 85–95% of control values at 41°C and to 85–95% of control fibroblasts at 37°C. However, fibroblasts from patients with the p.F352C (heterozygous) + p.V368I (homozygous) variant exhibited significantly decreased CPT II activity to 40–60%, and ATP production and fatty acid oxidation to 50–70% of control values at 41°C, and to control fibroblasts at 37°C, with further inactivation of CPT II activity at increasing temperatures. The effect of the attenuated CPT II activity on cellular production of ATP was slightly less than that on fatty acid oxidation.

## Discussion

Carnitine palmitoyltransferase II deficiency is the most common inborn errors of long-chain fatty acid oxidation and ATP generation, involving an increase in the concentrations of acylcarnitine in the serum [31–34]. In our previous data, we observed a transient and rapid increase of acylcarnitine ratio [(C_16:0_ + C_18:1_)/C_2_] to >0.09 in the serum during febrile convulsions that in the patients presenting with high risk influenza-associated encephalopathy, in the absence of previous episodes [24]. Because of the CPT II inactivation the rise in serum acylcarnitine probably caused by the presence of some disease causing mutations. However, these are not identical to the *CPT2* mutations observed in CPT II deficiency, which has a more severe phenotype.

Although a large proportion of CPT II deficiency patients exhibit phenotype of compound hetero/homozygous variations [26–30], the molecular mechanism of how the CPT II deficiency induces energy crisis has yet to be clarified. The genotype-phenotype correlation analysis of *CPT2* missense mutations has enabled the myopathic form of CPT II deficiency to be associated with “mild” mutations, while the null mutations resulting to truncation of mRNA or protein degradation are associated with the lethal neonatal forms [18, 30, 35–38]. Many disease causing mutations from inherited disorders effect on impair protein folding or reduce protein stability despite almost normal protein folding. In the present study, we analyzed three types of CPT II variations: p.V368I (heterozygous), p.V368I (homozygous), and p.F352C (heterozygous) + p.V368I (homozygous). Our data indicated that the fibroblasts of patients with these variants exhibited decreased enzyme activities, cellular β-oxidation, ATP generation, and ΔΨ_m_, with increased *Km* values for L-carnitine, thermal instability, short half-lives, and cellular apoptosis. p.F352C (heterozygous) + p.V368I (homozygous) fibroblasts showed a particularly prominent decrease in enzyme activity, β-oxidation, ATP generation, ΔΨ_m_, and lowered thermal stability, especially at 41°C and despite being located far from the acylcarnitine binding sites in the case of the p.F352C variant. The p.V368I variant is located near the acylcarnitine binding sites, though this appeared to have little effect on enzyme properties. Our work confirmed that CPT II variations have a dominant-negative effect on CPT II protein as shown previously in correlation researches between clinical metabolic and genotype data [18–26].

*CPT2* mutations have been reported to cause several metabolic disorders including general muscle damage, temperature changes, infections, disturbances of Na^+^/K^+^ ATPases, neuroleptic syndrome, excessive use of muscular force, and conditions related to lipid metabolism increases. Patients with these disorders require more energy than that is provided by glycolytic system, so rely on that provided by fatty acid oxidation pathway. Because CPT II is not a rate limiting enzyme for fatty acid β-oxidation and ATP product, the activity decrease does not correlate linearly with the fatty acid β-oxidation or ATP generation decrease. However, the thermolabile phenotype of *CPT2* polymorphism p.F352C demonstrates reduced CPT II enzyme activity.

**Fig 7:**
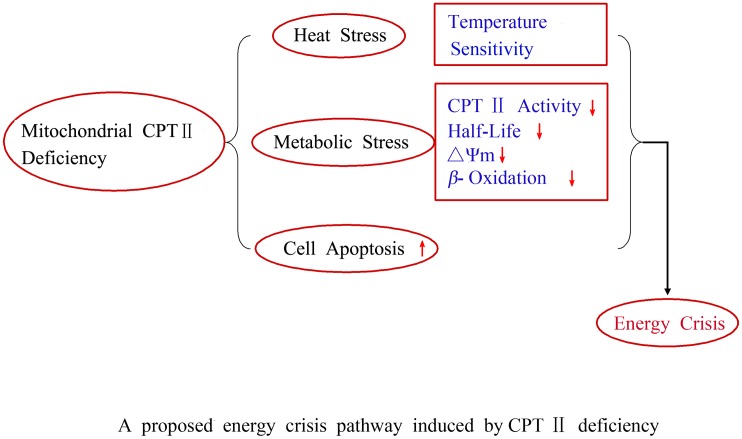
A proposed energy crisis pathway induced by CPT II deficiency.

Our current study characterized the enzymatic properties of CPT II variants found in patients with CPT II deficiency and defined the correlation between genotype and metabolism of β-oxidation, ATP production, and ΔΨm. The p.F352C (heterozygous) + p.V368I (homozygous) variant is associated with an energy crisis, especially at high temperature, and as such is suspected to be a risk factor for acute encephalopathy. The sudden-onset brain edema with clinical manifestation in patients supports the ATP threshold hypothesis in the etiology of this kind edema. The significantly reduced ATP levels and markedly reduced ΔΨm observed in the fibroblasts of this variant at 41°C suggests that such thermolabile compound variants of CPT II reduced fuel utilization in brain endothelial cells may be a cause of this kind acute brain edema during fever in the affected patients [39–41]. Moreover, the short half-lives and thermal instability of these CPT II variants may play an important role in decreasing ATP levels below the phenotypic threshold. These acquired short half lives, cell apoptosis, impaired mitochondrial fuel utilization, together with the serum acylcarnitine level rise that occurs during high fever, could be hypothesized as the main cause of energy crisis and multiple organ failure (Fig. 7).

Fibrates are hypolipidemic drugs that increase lipoprotein levels by up-regulating the mRNA expression of metabolism genes via the steroid—thyroid PPARa transcription factor interactions. Bezafibrate has been reported to increase *CPT2* mRNA expression and to normalize enzyme activity in cultured CPT II-deficient fibroblasts. CPT II-deficient patients administered bezafibrate also showed a normalization of fatty acid oxidation levels, and increased CPT II mRNA and protein levels [42–44]. Additional studies are currently underway to further examine the effects of fibrates on CPT II-deficient patients.

## Supporting Information

S1 Table Primers used for CPT2 variants sequence analysis. (DOC)

S2 Table Enzymatic properties of normal and patient CPT IIs. (DOC)
