# Supplementary material for: Abbreviated Half-Lives and Impaired Fuel Utilization in Carnitine Palmitoyltransferase II Variant Fibroblasts
Source: PLoS One. 2015 Mar 17;10(3):e0119936. doi: 10.1371/journal.pone.0119936 (PMC4364069; doi:10.1371/journal.pone.0119936)
Supplement: S1 Table — (DOC) [file pone.0119936.s001.doc]

**S1 Table. Primers used for *CPT2* variants sequence analysis**

Region Forward primer 　　　 Reverse primer Product size (bp)

Exon1-1 5’-ctgtgcagggttcagggtcattatatg-3’ 5’-cgatcctgggacggcgg-3’ 843

Exon1-2 5’-cctactagtgggcggggcctgtcagtgagc-3’ 5’-ggaaacgggttcactagaggagtcatgagtgactg-3’ 472

Exon2 5’-attaacctcttccatatactgtcagcc-3’ 5’-ccaccactacttgccagcct-3’ 201

Exon3 5’-catgaacctaaaaatcatgtattcccta-3’ 5’-cattatggagggctctgggag-3’ 221

Exon4 5’-gggacagcattaacattttatgttattt-3’ 5’-ccaagcactgaggacaagacc-3’ 1402

5’-cttcactgatgacaaggccagac-3’ 5’-tgtcatcagtgaagagttcatccc-3’

5’-ctctactgccgtccactttgagc-3’ 5’-cattaaaaaatctgagcactgccac-3’

5’-gccacctacgagtcctgtagca-3’ 5’-gcggatggtctcagtgcg-3’

5’-cctacctggtcaatgcgtatc-3’ 5’-catctaggcagagacagaacac-3’

Exon5 5’-ccttttccatcctgagactct-3’ 5’-agatctttgtgaggattaggaattaggt-3’ 1027

5’-aggcaaatccatcaaaagttaacttc-3’ 5’-tttcatgatgaggaagtgatggtag-3’

5’-ggcgacagagcgagactgtc-3’ 5’-cacccactggctacacaggc-3’

Reference sequences are GenBannk accession number NC_000001 for human CPT II genomic DNA. GenBank accession number NM_000098 for normal human CPT II.
