# Supplementary material for: Abbreviated Half-Lives and Impaired Fuel Utilization in Carnitine Palmitoyltransferase II Variant Fibroblasts
Source: PLoS One. 2015 Mar 17;10(3):e0119936. doi: 10.1371/journal.pone.0119936 (PMC4364069; doi:10.1371/journal.pone.0119936)
Supplement: S2 Table — (DOC) [file pone.0119936.s002.doc]

**S2 Table. Enzymatic properties of normal and patient CPT IIs**

Variant Specific activity *Vmax*  *Km*

(nmol/min/mg) (nmol/min/mg) (mM)

Normal 0.28 ± 0.03 0.72 ± 0.03 1.85 ± 0.03

V368I (Hetero) 0.26 ± 0.03 0.70 ± 0.03 1.90 ± 0.03

V368I (Homo) 0.25 ± 0.03 0.70 ± 0.03 1.90 ± 0.03

F352C (Hetero) + V368I (Homo) 0.15 ± 0.03 0.57 ± 0.03 2.68 ± 0.03

CPT II activities and their kinetic properties were measured as described under “Experimental Procedures”. Data are presented as mean ± S.D. of five independent experiments with different protein preparations.
